# Development and validation of a nomogram to predict overall survival and cancer-specific survival in patients with primary intracranial malignant lymphoma: A Retrospective study based on the SEER database

**DOI:** 10.3389/fonc.2022.1055046

**Published:** 2023-01-09

**Authors:** Ziyue Yang, Zhenfen Li, Chunmeng Fu, Yuanyuan Zhu, Ying Lin, Ying Deng, Ning Li, Fang Peng

**Affiliations:** ^1^ Department of Blood Transfusion, Xiangya Hospital, Central South University, Changsha, Hunan, China; ^2^ National Health Commission (NHC) key Laboratory of Cancer Proteomics, Xiangya Hospital, Central South University, Changsha, Hunan, China; ^3^ Department of Nuclear Medicine, Xiangya Hospital, Central South University, Changsha, China; ^4^ Department of Scientific Research Management, Ningxiang People’s Hospital, Hunan University Traditional Chinese Medicine, Ningxiang, Changsha, Hunan, China

**Keywords:** primary intracranial malignant lymphoma, neurological tumors, diffuse large B lymphoma, SEER database, nomogram

## Abstract

**Introduction:**

Primary intracranial malignant lymphoma (PIML) is a rare form of lymphoma that most often occurs in the brain and has an extremely low 5-year survival rate. Although chemotherapy and radiotherapy are widely used in the clinical management of PIML, the choice of treatment regimen and the actual circumstances of patients remain challenges when assessing survival rates in different patients.

**Methods:**

Considering this, we obtained clinical treatment and survival information from the Surveillance, Epidemiology, and End Results database (SEER) on patients with lymphoma, the primary site of which was the brain, and performed statistical analyses of the demographic characteristics. Survival analyses were performed using the Kaplan–Meier method, and univariate and multivariate Cox proportional hazards regression analyses were performed to identify independent prognostic factors.

**Result:**

We identified age, pathology, the Ann Arbor stage, and treatment as the risk factors affecting patient prognosis. The areas under the curve (AUCs) for overall survival at 1, 3, and 5 years were 0.8, 0.818, and 0.81, respectively. The AUCs for cancer-specific survival at 1, 3, and 5 years were 0.8, 0.79, and 0.79. The prediction ability in the development and verification cohorts was in good agreement with the actual values, while we plotted the clinical decision curves for the model, suggesting that the nomogram can provide benefits for clinical decision-making.

**Conclusion:**

Our model provides a prognostic guide for patients with PIML and a reliable basis for clinicians.

## Introduction

Primary intracranial malignant lymphoma (PIML) is an uncommon form of lymphoma, accounting for 1% of all non-Hodgkin’s lymphomas (1). Its incidence has increased over the last 30 years and is closely related to the immune function of patient (2). PIML has become the most common neurological tumor because of the increase in patients with acquired immunodeficiency syndrome and the use of immunosuppressive drugs following transplantation (3). Although PIML is relatively rare, it is more common than the secondary spread of primary extracranial lymphoma (4).

PIML is treated with a combination of chemotherapy, radiotherapy, and surgery in clinical practice (5). Of these, chemotherapy is the most conventional treatment (6), and experts in the field agree that high doses of methotrexate are the backbone of multimodal therapy (7), including other chemotherapeutic agents. However, there are many controversies regarding the treatment of PIML. The impact of surgical resection on PIML remains controversial, with one clinical study suggesting that for intracranial lymphoma, surgical resection improves PFS (progression-free survival) but not OS (1). Other controversies include the optimal upfront chemotherapy regimen, the status of radiotherapy, the risks and benefits of surgical treatment, and treatment involving the cerebrospinal fluid space (8).

To explore the impact of different demographic characteristics and treatment on the prognosis of patients with PIML, we performed a retrospective analysis of data corresponding to patients with PIML based on the Surveillance, Epidemiology and End Result (SEER) database (9). SEER database, supported by a project of the National Cancer Institute (NCI), is a real-world database of clinical oncology literature that has been widely used for epidemiological investigations and statistical analyses (10), especially for a rare tumor type such as PIML. With a total of 1245 patients diagnosed with lymphoma of the brain origin between 1975 and 2019, this is the most comprehensive retrospective study to analyze clinicopathological features. Using these data and establishing a nomogram model that predicts OS and CSS (Cancer Specific Survival), which will provide an excellent reference for future clinical decisions.

## Method

### Data acquisition

**Figure 1:**
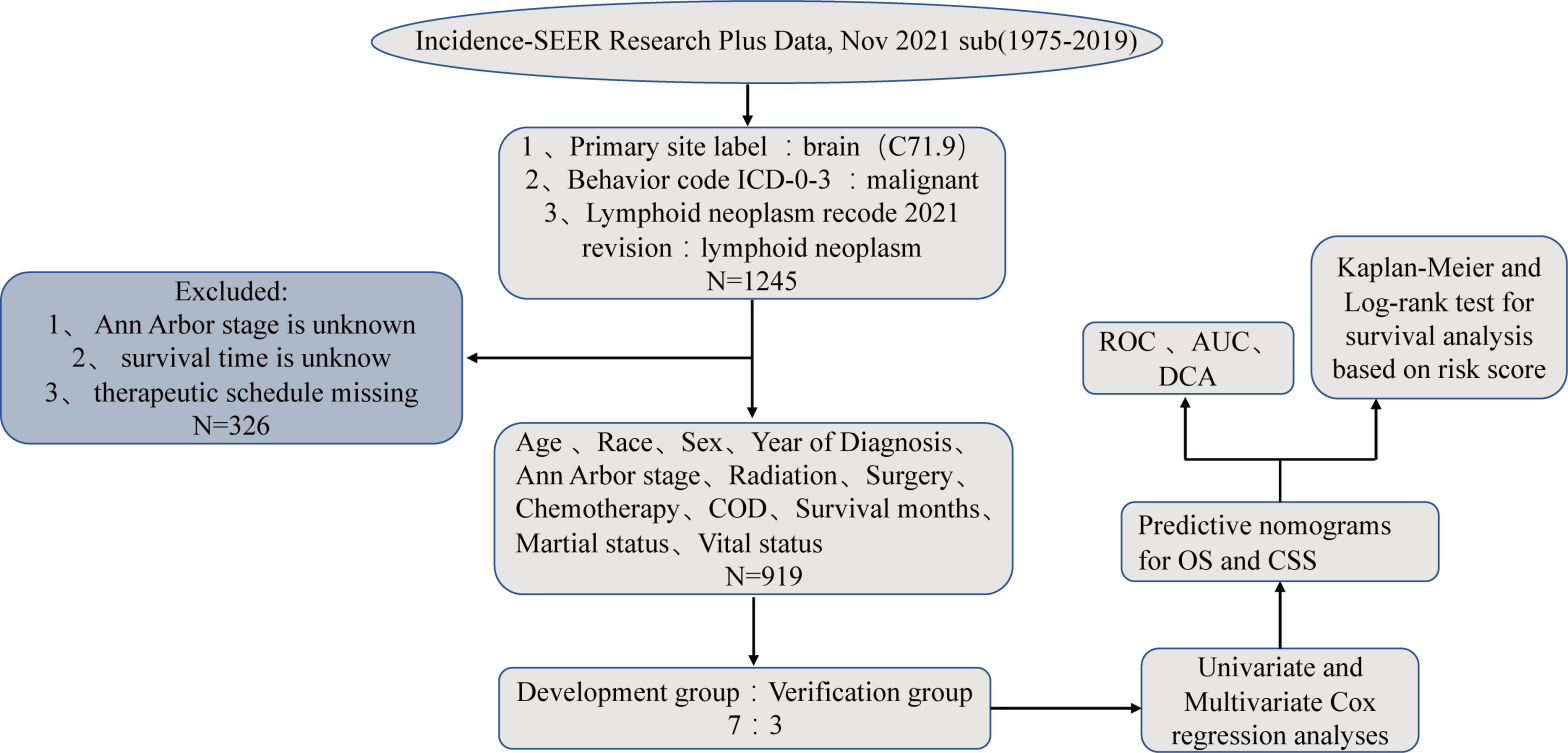
The flowchart of this study which was including and dividing patients.

The SEER Program of the NCI contains retrospective data on the demographic characteristics, disease classification, pathological features, and treatment of tumors in patients from different states in the USA (10). In its official data retrieval SEER* Stat software, the “Incidence-SEER Research Plus Data, Nov 2021 sub (1975–2019)” dataset corresponded to patients with PIML from 1975-2019. The primary site label identified the brain (code C71.9), and the behavior code ICD-0-3 was used to identify the malignance. The disease was identified as a lymphoid neoplasm after the 2021 revision. We collected information on sex, age, the time of diagnosis, the primary tumor site, pathological staging of the lymphoma, radiotherapy, chemotherapy, surgical treatment, survival time, marital status, the cause of death, and survival status. A total of 919 patients were included after screening based on the following exclusion criteria (1): Ann Arbor stage information was unknown (2), treatment information was unknown (3), survival time was unknown. The graphical abstract of this study is presented in **Figure 1**.

### Statistical analysis

All statistical analyses were performed using R Studio (version 4.1.2) and SPSS (version 36.0). First, the 919 samples were randomly divided in a 7:3 ratio into development and verification groups using the sample statement in R Studio (11). These groups were used to develop and validate the model. Statistical analysis between the two groups was performed using the R package “CompareGroups,” and chi-square tests were used for comparisons between the groups. The R package “survival,” “rms,” “survivalROC,” “survminer,” and “ggplot2” were used for survival analysis and to develop nomogram models and plot Kaplan–Meier (KM) curves.

OS (Overall survival) and CSS (cancer-specific survival) are commonly used indicators to assess the prognostic status of tumor patients. First, OS and CSS were used as the outcome variables. Using the KM method, OS and CSS were calculated using SPSS software. We obtained the Cox regression model parameters using maximum likelihood estimation with the help of the partial likelihood function. We included all variables in the development cohort in the univariate Cox regression analysis separately, and *P* values < 0.05 were considered statistically significant. Variables that were statistically and clinically significant were included in the multivariate Cox analysis, while hazard risk ratios were calculated, with a value > 1 being considered a risk factor for survival. The independent prognostic factors affecting OS and CSS in patients with PIML were identified. A nomogram prediction model was developed based on the established Cox regression results. All the above was also verified in the verification cohort.

A risk score formula was constructed based on multivariate Cox regression. All the patients were divided into high and low risk groups according to the risk scores, and the probability of poor survival was quantified for each patient. A nomogram model was constructed based on the multivariate Cox regression analysis. To further evaluate the predictive performance of the nomogram model, the area under the subject working characteristic curve (AUC) and correction curve for OS and CSS at 1, 3, and 5 years were calculated (11). A higher AUC and smoother correction curves indicated that the models had a greater ability to predict patient prognosis (12). Decision curve analysis (DCA) also demonstrated that our models could deliver greater patient benefits (13). Finally, the KM method was used to plot survival curves with different variables.

## Result

### Patients’ characteristics

A total of 919 patients diagnosed with lymphoma between 1975 and 2019 with the primary site in the brain and documented survival time and clinical staging were included in the study. **Table 1** summarizes the demographic and clinical characteristics of all the patients. The patients were randomly distributed into a developmental group (n = 643) and a verification group (n = 276), and the chi-square test was used to confirm the significant internal differences between the two groups. The International Prognostic Index (IPI) stage of lymphoma scores 60 years as 0 points and age >60 years as 1 point (14). In this study, all patients were classified according to the IPI scoring system using the age of 60 years as the cutoff value. Of these, 573 patients (62%) were younger than 60 years at the time of diagnosis. Caucasian ethnicity predominated (n = 735, 80%), and there were 609 (66%) male patients.

**Table 1:** The demographic and clinical features of patients with PIML.

	Total	Development Cohort	Verification Cohort	*P Value*
	N=919	N=643(70%)	N=276 (30%)	
Age				0.202
<60	573(62.3%)	410 (63.8%)	163 (59.1%)	
>=60	346(37.7%)	233 (36.2%)	113 (40.9%)	
Race				0.981
Black	103(11.2%)	71 (11.0%)	32 (11.6%)	
Other	80(8.8%)	56 (8.71%)	24 (8.70%)	
Unknown	1(0.10%)	1 (0.16%)	0 (0.00%)	
White	735(80.0%)	515 (80.1%)	220 (79.7%)	
Sex:				0.005
Female	310(33.7%)	198 (30.8%)	112 (40.6%)	
Male	609(66.3%)	445 (69.2%)	164 (59.4%)	
Year of Diagnosis:				0.005
1989-1983	66(7.2%)	49 (7.62%)	17 (6.16%)	
1990-1999	398(43.3%)	292 (45.4%)	106 (38.4%)	
2000-2009	265(28.8%)	189 (29.4%)	76 (27.5%)	
2010-2015	190(20.8%)	113 (17.6%)	77 (27.9%)	
Histology:				0.387
DLBCL	462(50.3%)	316 (49.1%)	146 (52.9%)	
HL	2(0.2%)	2 (0.31%)	0 (0.00%)	
Lymphoid neoplasm*	346(37.6%)	252 (39.2%)	94 (34.1%)	
NHL	109(11.9%)	73 (11.4%)	36 (13.0%)	
Ann Arbor stage:				0.921
Stage I	729(79.3%)	512 (79.6%)	217 (78.6%)	
Stage II	11(1.2%)	7 (1.09%)	4 (1.45%)	
Stage III	6(0.7%)	4 (0.62%)	2 (0.72%)	
Stage IV	173(18.8%)	120 (18.7%)	53 (19.2%)	
Surgery:				0.977
NO	767(83.4%)	536 (83.4%)	231 (83.7%)	
YES	152(16.6%)	107 (16.6%)	45 (16.3%)	
Radiation:				0.303
NO	465(50.6%)	333 (51.8%)	132 (47.8%)	
YES	454(49.4%)	310 (48.2%)	144 (52.2%)	
Chemotherapy:				0.974
No/Unknown	547(59.5%)	382 (59.4%)	165 (59.8%)	
Yes	372(40.5%	261 (40.6%)	111 (40.2%)	
COD:				0.342
Alive	98(10.7%)	66 (10.3%)	32 (11.6%)	
NHL	371(40.4%)	252 (39.2%)	119 (43.1%)	
other	450(49.0%)	325 (50.5%)	125 (45.3%)	
Marital status:				0.141
Divorced	75(8.2%)	49 (7.62%)	26 (9.42%)	
Married	387(42.1%)	275 (42.8%)	112 (40.6%)	
Separated	6(0.7%)	3 (0.47%)	3 (1.09%)	
Single (never married)	361(39.3%)	262 (40.7%)	99 (35.9%)	
Unknown	21(2.3%)	11 (1.71%)	10 (3.62%)	
Unmarried or Domestic Partner	1(0.10%)	1 (0.16%)	0 (0.00%)	
Widowed	78(7.3%)	42 (6.53%)	26 (9.42%)	
Status:				0.63
Alive	98(10.7%)	66 (10.3%)	32 (11.6%)	
Dead	821(89.3%)	577 (89.7%)	244 (88.4%)	
Overall Survival				
1-year	270(29.5%)			
3-year	190(21.0%)			
5-year	131(16.1%)			
Cancer-specific Survival				
1-year	225(27.5%)			
3-year	156(19.3%)			
5-year	104(14.6%)			

*Lymphoid neoplasm is a group of malignant neoplasm originated from all lymphocytes.

Diffuse large B-cell lymphoma was the most common histological subtype, with 462 cases (50%), which is sufficient to establish that the most common pathological type of intracranial lymphoma is the same as the systemic lymphoma (15). This was followed by lymphoid tumors in 346 cases (38%). Hodgkin’s lymphoma was the least common, with only 2 cases (0.2%). For lymphoma, Ann Arbor staging guided clinical diagnosis and treatment was used, with stages I and IV accounting for the majority of the 919 patients included in the study, 729 (80%) and 173 (19%), respectively. For treatment, 152 (17%) patients underwent surgery, with some of the remaining patients treated for surgery due to patients’ refusal and the lack of indications for surgery. Radiotherapy and chemotherapy were administered to 454 (49%) and 372 (40%) patients, respectively. Due to the limitations of the database, it was not possible to know about the specific treatment regimens of the patients or the chemotherapeutic drugs used.

**Figure 2:**
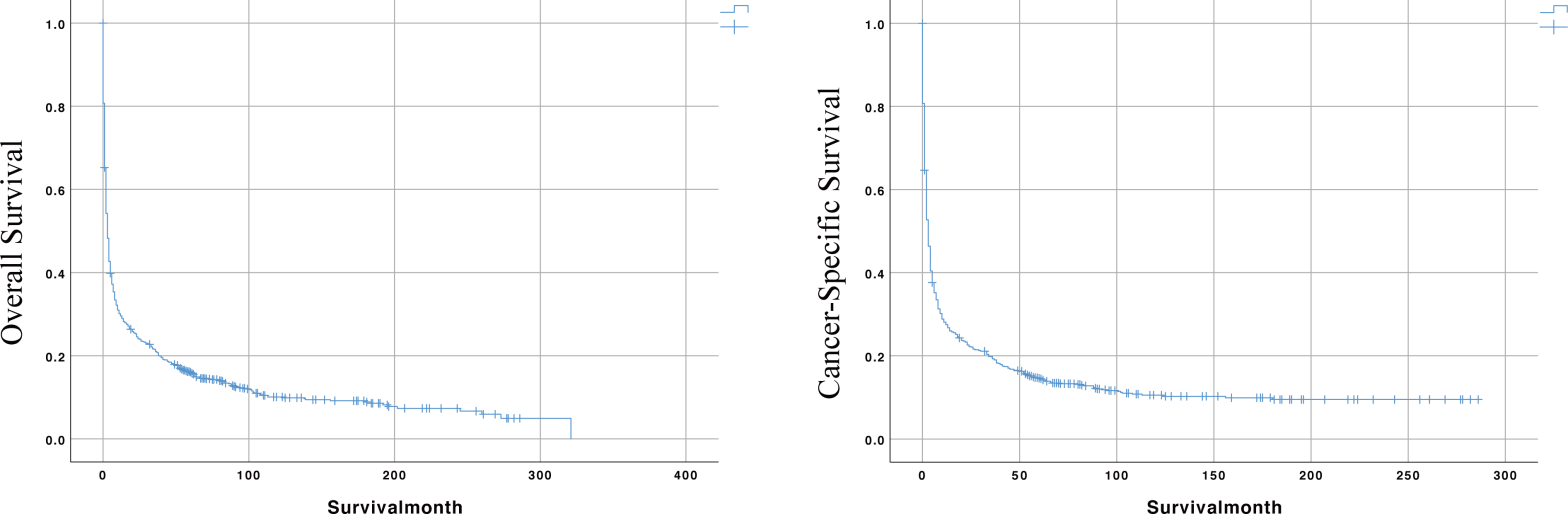
The Kaplan-Meier (KM) curves for predicting the OS and CSS of PIML patients in the whole cohort.

Meanwhile, the median survival time for the entire cohort was 3 months. As shown in the survival curves in **Figure 2**, the OS rates for the entire cohort were 29.5%, 21.0%, and 16.1% at 1, 3, and 5 years. The CSS rates were 27.5%, 19.3%, and 14.6% at 1, 3, and 5 years, respectively.

### Survival analysis of OS and CSS

Univariate and multivariate Cox regression analyses were performed to predict prognostic factors affecting OS and CSS. First, all variables in the development cohort, including age, sex, race, the time of diagnosis, the type of pathology, the Ann Arbor stage, radiotherapy, surgical treatment (other than biopsy), chemotherapy, and marital status were included in the statistical analysis. The results shown in **Table 2** indicate that age was a potential risk factor affecting OS. Marital status is also a potential prognostic factor. In addition, the pathological type of Hodgkin’s lymphoma, surgery, and chemotherapy were significantly associated with OS. However, our results showed that radiotherapy was not significantly associated with OS.

**Table 2:** The result of Univariate and Multivariate COX regression analyses of OS and CSS in development group.

	Overall Survival using COX regression						Cancer-Specific Survival using COX regression					
	Univariate Analysis (Development)			Multivariate Analysis (Development)			Univariate Analysis (Development)			Multivariate Analysis (Development)		
	HR	95% CI	*P value*	HR	95%CI	*P value*	HR	95%CI	*P value*	HR	95%CI	*P value*
Age												
<60	ref			ref			ref			ref		
>=60	1.766	1.491-2.092	4.65e-11 ***	1.73282	1.4514-2.0688	1.2e-09 ***	0.9427	0.7904-1.124	0.512			
Sex												
Female	ref						ref					
Male	1.055	0.8829-1.26	0.556				1.538	1.271-1.861	9.44e-06 ***	1.1581	0.9264-1.4477	0.19739
Race												
Black	ref						ref					
Other	1.1644	0.89644-1.513	0.254				0.6149	0.4644-0.8143	0.000688 ***	0.872	0.6504-1.1691	0.35986
Unknown	0.6803	0.09432-4.906	0.702				/	/	/	/	/	/
White	1.061	0.72963-1.543	0.756				0.4892	0.3264-0.7331	0.000532 ***	0.7858	0.5147-1.1997	0.26418
Year of Diagnosis												
1989-1983	ref						ref					
1990-1999	0.881	0.6473-1.199	0.42				1.1361	0.8113-1.5910	0.457764	1.1478	0.8074-1.6319	0.44247
2000-2009	0.9062	0.6565-1.251	0.549				0.5657	0.3940-0.8123	0.002026**	0.8158	0.5572-1.1943	0.29509
2010-2015	1.2022	0.8530-1.694	0.293				0.48	0.3282-0.7022	0.000156***	0.7686	0.5117-1.1545	0.20483
Histology												
NHL	ref			ref			ref			ref		
DLBCL	0.8227	0.2046-3.3077	0.78339	1.0005	0.2461-4.0669	0.99945	1.4161	0.1985-10.105	0.729	1.5833	0.2123-11.8074	0.65398
Lymphoid neoplasm	0.8143	0.6832-0.9707	0.02192	1.1401	0.9261-1.4037	0.21648	1.7262	1.4346-2.077	7.34e-09***	1.0552	0.8609-1.2932	0.60494
HL	1.4194	1.0899-1.8485	0.00936 **	1.325985	1.0124-1.7368	0.04045 *	0.9117	0.6677-1.245	0.561	0.883	0.6391-1.2200	0.45065
Ann Arbor stage												
Stage I	ref						ref					
Stage II	1.7901	0.8481-3.778	0.127				0.5465	0.2262-1.320	0.179			
Stage III	0.8278	0.3092-2.216	0.707				0.8944	0.2872-2.785	0.847			
Stage IV	0.949	0.7688-1.171	0.626				1.0607	0.8468-1.329	0.608			
Radiation												
NO	ref						ref					
YES	0.861	0.7311-1.014	0.0728				0.9552	0.8783-1.248	0.609			
Surgery												
NO	ref			ref			ref			ref		
YES	0.5511	0.4372-0.6947	4.57e-07 ***	0.654283	0.4963-0.8626	0.00263 **	0.5614	0.4365-0.7222	7.03e-06 ***	0.6895	0.5280-0.9004	0.00633 **
Chemotherapy												
No/Unknown	ref			ref			ref			ref		
Yes	0.3635	0.3048-0.4336	<2e-16 ***	0.4078	0.3374-0.4930	< 2e-16 ***	0.3474	0.2875-0.4197	<2e-16 ***	0.4494	0.3572-0.5653	8.42e-12 ***
Marital status												
Single	ref			ref			ref			ref		
Married	0.7795	0.5648-1.076	0.1298	0.8982	0.6471-1.2468	0.52114	0.8492	0.6022-1.197	0.35115	1.1182	0.7783-1.6066	0.54563
Separated	2.0409	0.6324-6.586	0.2327	1.1564	0.3551-3.7661	0.80938	1.9232	0.6862-5.390	0.2136	1.3042	0.4549-3.7388	0.62115
Unknown	1.4244	1.0319-1.966	0.0315*	1.1808	0.8508-1.6388	0.32039	1.6273	1.1561-2.291	0.00525 **	1.1999	0.8370-1.7201	0.32125
Widowed	1.054	0.5304-2.095	0.8807	1.0101	0.5069-2.0129	0.97728	1.2469	0.6385-2.435	0.51813	1.2103	0.6127-2.3907	0.58271
Divorced	1.5957	0.2195-11.599	0.6443	1.8864	0.2563-13.8841	0.53317	1.6645	0.2283-12.138	0.61523	2.0404	0.2694-15.4506	0.48995
Unmarried or Domestic Partner	1.2492	0.8183-1.907	0.3026	1.2287	0.8011-1.8845	0.34534	1.3386	0.8582-2.088	0.19856	1.2886	0.8176-2.0309	0.27459

*P<0.05, **p<0.005, ***p<0.0005.

Variables that were statistically significant in the univariate Cox analyses were then included in the multivariate Cox regression analysis. The data showed that age was an independent risk factor affecting the prognosis of patients, with patients 60 years or older having a poor prognosis. The pathology type of Hodgkin’s lymphoma was also an independent factor affecting OS, with patients with Hodgkin’s lymphoma having worse OS. Chemotherapy and surgery were independent prognostic factors, with the risk of death being 0.41 times higher for those who received chemotherapy than for those who did not, and 0.65 times higher for those who received surgery than for those who did not, suggesting that patients treated with chemotherapy and surgery had better OS. Cox regression analysis showed that the Ann Arbor stage was not a prognostic factor. This may be related to the distribution of the data, as lymphoma is an insidious malignancy with high heterogeneity, and all data were distributed closer to stages I and IV. Nonetheless, the Ann Arbor stage remains the preferred assessment criterion in clinical decision-making.

**Figure 3:**
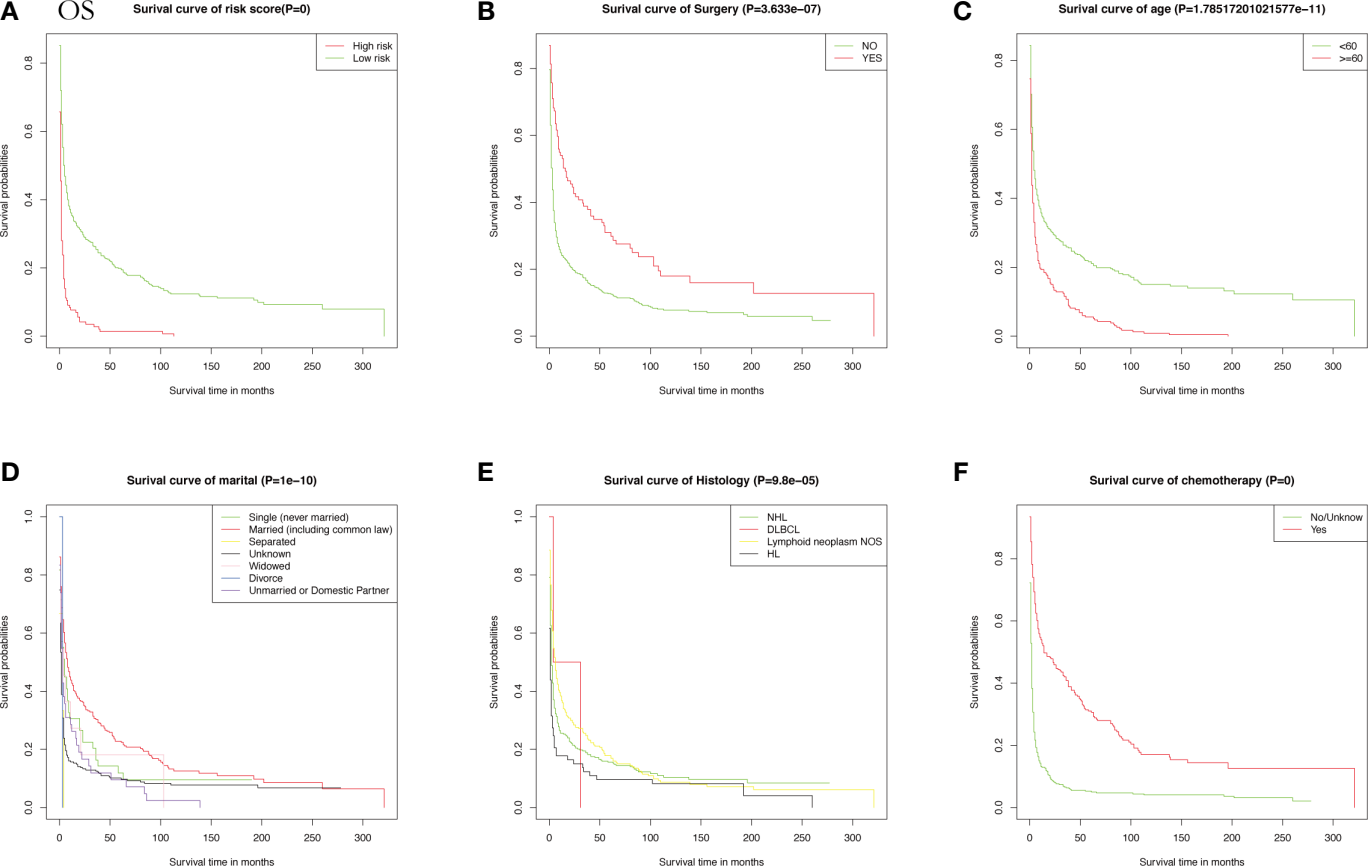
The Kaplan-Meier (KM) method was performed to calculate the OS classified by clinical pathologic factors for patients with PIML. The clinical pathologic factors including risk score **(A)**, Surgery **(B)**, Age **(C)**, Marital status **(D)**, Histology **(E)**, Chemotherapy **(F)**.

**Figure 4:**
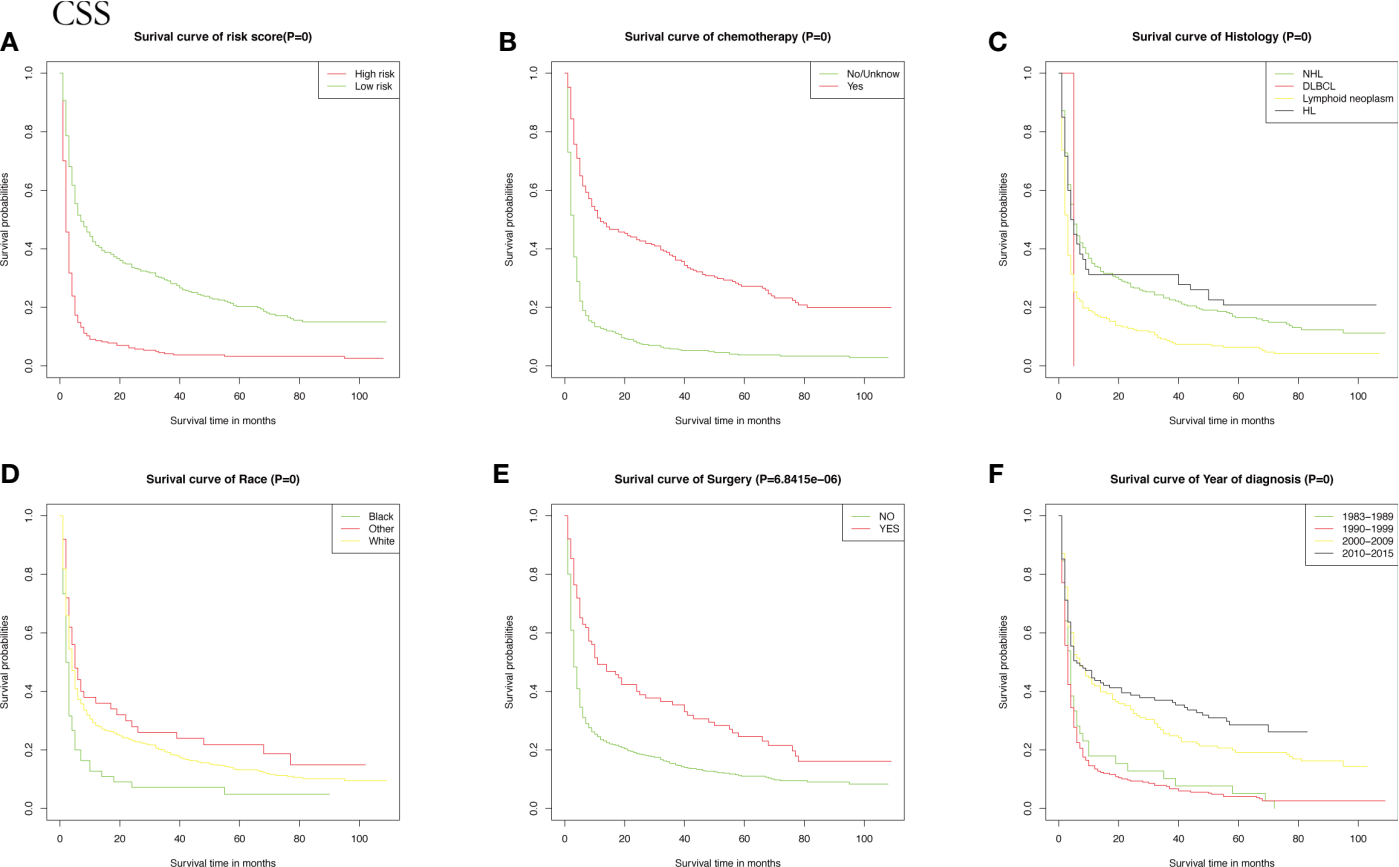
The Kaplan-Meier (KM) method was performed to calculate the CSS classified by clinical pathologic factors for patients with PIML. The clinical pathologic factors including risk score **(A)**, Chemotherapy **(B)**, Histology **(C)**, Race **(D)**, Surgery **(E)**, Year of diagnosis **(F)**.

Meanwhile, CSS is one of the most important predictors of prognosis in patients with malignancy. The impact of the 10 aforementioned variables on CSS was further explored. In contrast to OS, the results of univariate Cox analysis showed that age did not have the potential to influence CSS. Sex and age were able to influence CSS, and the time to diagnosis was a potential predictor of CSS. As previously described, these statistically significant differences in the univariate analysis were further included in the multivariate Cox analysis, which showed that age and sex had a *P* value greater than 0.05 and failed to show independent predictive power. Like OS, surgery (*P* = 0.00633) and chemotherapy (*P* = 8.42e-12) remained highly significant independent prognostic factors. In summary, both statistically and in clinical practice, treatment significantly influenced the prognostic survival of patients, but statistical analysis showed that radiotherapy did not demonstrate superiority. Next, the KM method was used to calculate the probability of survival for OS (**Figure 3**) and CSS (**Figure 4**) in PIML, and all cohorts were categorized by variables, showing a significant correlation with prognosis.

### Establishment and evaluation of prognostic nomogram

**Figure 5:**
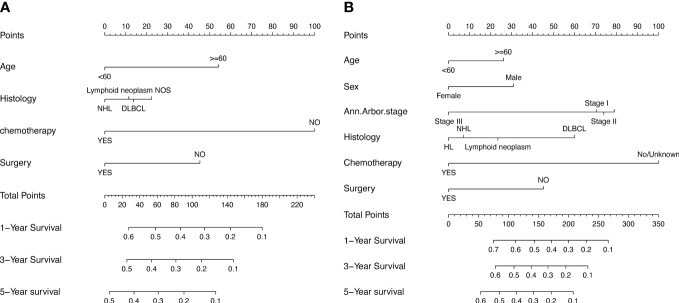
The prognostic nomogram models to predict 1-year, 3-year, and 5-year of OS and CSS for patients *via* development cohort **(A, B)**.

**Figure 6:**
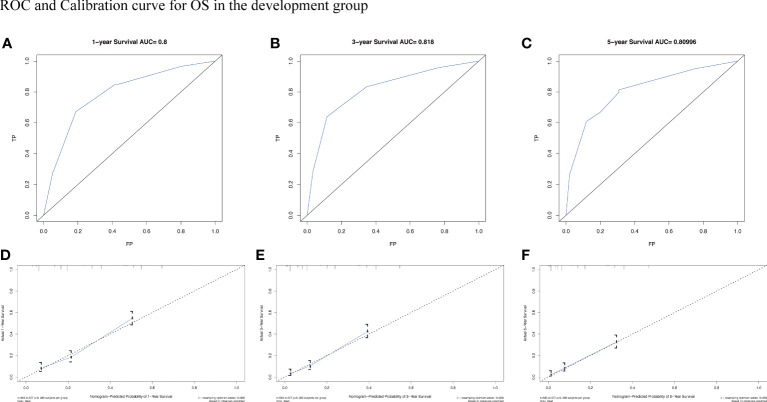
The AUC **(A-C)** and calibration curve **(D-F)** of 1-year, 3-year, and 5-year to assess the performance of predictive models for OS in the development group.

**Figure 7:**
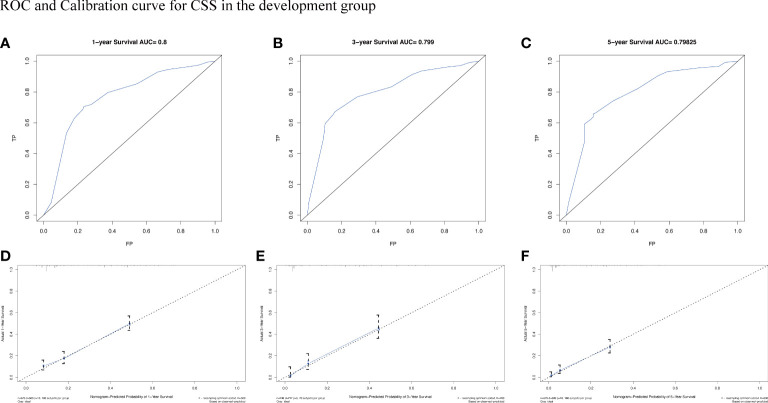
The AUC **(A-C)** and calibration curve **(D-F)** of 1-year, 3-year, and 5-year to assess the performance of predictive models for CSS in the development group.

**Figure 8:**
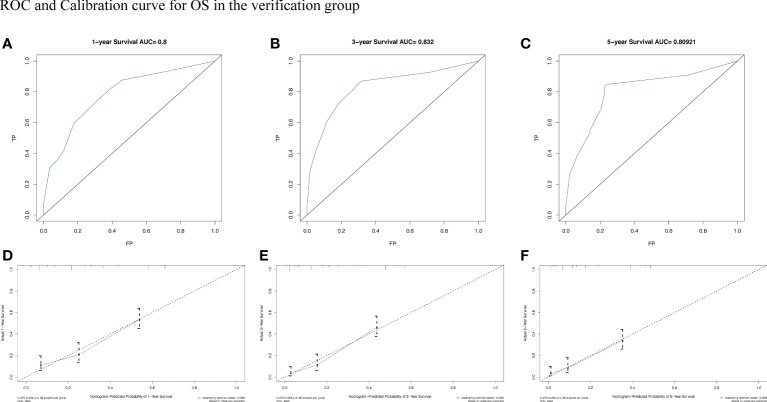
The AUC **(A-C)** and calibration curve **(D-F)** of 1-year, 3-year, and 5-year to assess the performance of predictive models for OS in the verification group.

**Figure 9:**
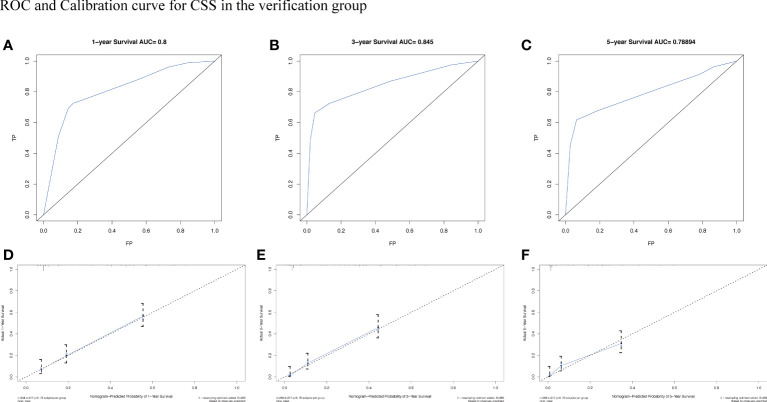
The AUC **(A-C)** and calibration curve **(D-F)** of 1-year, 3-year, and 5-year to assess the performance of predictive models for CSS in the verification group.

**Figure 10:**
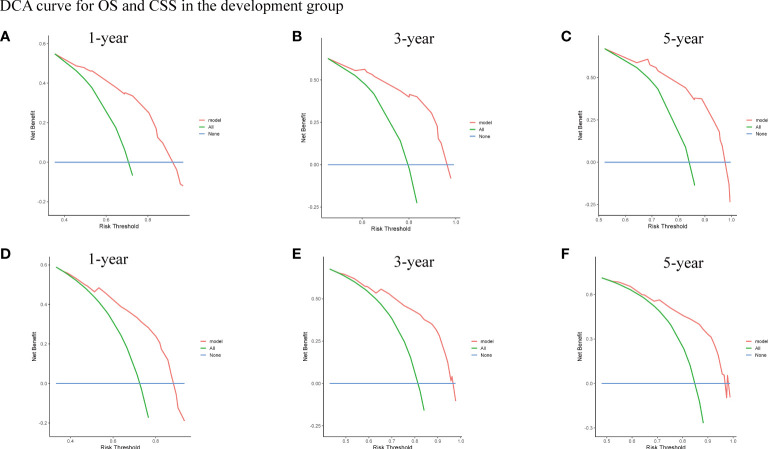
The DCA curve for OS **(A-C)** and CSS **(D-F)** of 1-year, 3-year, and 5-year in the development group.

To assess the survival probability in individual patients, the nomogram models for predicting patients’ OS and CSS were developed in the development cohort. Factors of significant prognostic and clinical significance were screened using multivariate Cox regression analysis, including age, the type of pathology, treatment, and Ann Arbor staging (**Figure 5**). Detailed scores for each factor are shown in the nomogram, and by calculating these scores against the total score on the bottom axis, OS and CSS could be predicted at 1, 3, and 5 years. To verify the feasibility of these models, receiver-operating characteristic curves were used to assess the accuracy of the nomogram models. The AUC values for OS at 1, 3, and 5 years were 0.8, 0.818, and 0.81. In the development cohort (**Figure 6**), and the AUC values for CSS were 0.8, 0.799, and 0.798, respectively (**Figure 7**). These data demonstrated the predictive power of the nomogram based on the development cohort. Next, the effectiveness of the nomogram was validated in the verification cohort to further demonstrate its reliability, with AUC values of 0.8, 0.832, and 0.809 for OS (**Figure 8**) and 0.8, 0.845, and 0.788 for CSS (**Figure 9**) in the verification cohort at 1, 3, and 5 years, respectively, demonstrating the potential ability of the established nomogram to predict 1-, 3- and 5-year survival rates in patients with PIML (**Figure 10**). Analysis of the AUC and calibration curves in these validated cohorts showed consistent results in the development cohort, and these results confirmed the strong relevance and value of the multi-established nomogram in predicting the prognosis of patients with intracranial lymphoma in clinical practice. The model built by the nomogram was further subjected to DCA, a simple method for evaluating clinical prediction models. The data showed that the model built according to the nomogram yielded good benefits, allowing patients to achieve higher benefits for the same risk.

Next, risk scores for patients with intracranial lymphoma were quantified based on the patients’ clinicopathological factors, age, sex, type of pathology, and treatment. To further illustrate whether risk scores could be considered independent prognostic factors, we determined the median risk score as a basis for classifying high-risk subgroups and low-risk wind resistance, with KM curves showing better survival rates in the low-risk group.

## Discussion

To our knowledge, PIML is a relatively rare intracranial malignancy that accounts for approximately 1.5% of all primary intracranial tumors (16). PIML can initially present with optic nerve damage, increased intracranial pressure, a high degree of malignancy, a specific release site, and a poor prognosis (17). The prognosis of this rare type of lymphoma, in which non-Hodgkin’s lymphoma is the main type, has improved over the last decade due to the use of high doses of methotrexate (18). However, the prognosis of PIML depends on several other factors, such as the time of diagnosis, histological and biological characteristics of the tumor, and the appropriateness of the treatment (19). In conclusion, after more than a decade of development, the prognosis of PLML remains unsatisfactory (20). Routine physical examinations are unable to assess intracranial conditions, and most intracranial abnormalities detected on magnetic resonance imaging, as well as their specific anatomical location, prevent clinicians from obtaining a specifically determined (21). Therefore, there is a need to explore sensitive and specific tumor markers and diagnostic methods. Owing to the rarity of intracranial lymphoma, there are only a few related clinical studies (22). According to the literature, there are no randomized clinical trials for recurrent, refractory PIML, and no prognostic models for PIML have been developed (23). To better understand the clinical demographic characteristics and risk factors of PIML, we investigated the largest sample of data corresponding to patients with PIML retrieved from the SEER database, and these data were used to develop and validate prognostic OS and CSS nomograms for patients with PIML. These models can be applied in clinical practice to provide advice to physicians when making clinical decisions.

This study included 919 patients who were diagnosed with lymphoma between 1975 and 2019 with an intracranial primary site and documented survival time and clinical staging. Detailed data on demographic characteristics were obtained and statistically analyzed, with OS rates of 29.5%, 21.0%, and 16.1% at 1, 3, and 5 years, respectively, for the entire cohort. Univariate Cox regression analysis was performed to identify potential risk factors, and multivariate Cox regression analysis was performed to identify independent prognostic factors. The patients were categorized according to IPI staging using a cut-off of 60 years of age, with 62.3% of patients <60 years. Age was one of the most notable risk factors for prognosis in patients with PIML; patients >60 years had worse OS, although 60 years of age was statistically insignificant for CSS based on the results. In addition, there appeared to be no statistically significant difference in the effect of sex on intracranial lymphoma, with a potentially significant difference in incidence by sex. Meanwhile, the pathology was consistent with that of lymphoma, with diffuse large B-cell type remaining the most common type, whereas patients with a pathological type of Hodgkin’s lymphoma had a worse prognosis, in line with the results of previous studies (24).

Ann Arbor staging was not significant based on the statistical analysis of this study. This may be strongly related to the distribution of data, with most patients (98.1%) having stages I and IV, which could have biased the results of the regression analysis. This reflects the following two extremes in the staging of patients with PIML: early onset of symptoms causing discomfort, leading to earlier medical intervention, or early onset of no obvious symptoms, and an advanced stage by the time of the first medical examination (25). However, the results of the statistical analysis do not negate the role of Ann Arbor staging in guiding clinical practice.

Meanwhile, treatment options are the most important factors affecting the prognosis of patients with PIML (26),and patients who have not undergone systemic treatment generally have a worse survival time. Management of patients with PIML included surgical tumor resection (other than biopsy), chemotherapy and radiation therapy. This multimodal treatment, including chemotherapy and aggressive surgery can convey a survival advantage in patients with PIML (27).However, radiotherapy does not show a unique superiority over chemotherapy and surgery, contrary to our conventional understanding (28).Patients treated with chemotherapy and surgery generally have longer life expectancy (29). These data confirm the importance of chemotherapy in the treatment regimen for PIML. However, owing to the limitations of the database, it is not possible to obtain information on the specific options for treatment, including surgery, the drug regimens for chemotherapy, and the choice of dose and irradiation target area for radiotherapy, all of which largely influence the patient outcomes (30).

A nomogram to predict patient prognosis was further developed based on a risk-based coefficient. Given the importance of age for patient staging, we included age in the creation of the nomogram. Firstly, time-dependent receiver-operating characteristic curves indicated that the nomogram had high sensitivity and specificity. Second, the small deviation from the reference line demonstrated the high reliability of the established nomogram, and the DCA curve showed that the nomogram contributed to better clinical decision-making (31).

For lymphomas of the central nervous system, MRI (magnetic resonance imaging) is the diagnostic method of choice. The T2-weighted (T2W) signal usually indicates intracranial edema and lesions of vascular origin, while the T1-weighted (T1W) signal shows intracranial parenchymal lesions or occupancies (32). For patients with PIML, there is no substitute for MRI in assessing the anatomical localization of the tumor, tumor size, and edema of the brain parenchyma. Meanwhile, positron emission tomography combined with computed tomography (PET/CT) is increasingly being demonstrated in clinical trials as the center of gravity in the evaluation of lymphoma. Gradually, a diagnostic approach with MRI and PET/CT as the core has been developed. In particular, the sensitivity of PET/CT has increased dramatically in patients with lymphoma involving bone marrow involvement (33). However, a subset of lymphoma patients exhibits low ^18^F fluorodeoxyglucose (FDG) avidity (34). Also, PET/CT based staging is prognostically instructive in most clinical explorations, suggesting that imaging can give direct evidence in tumor morphology, infiltration, and metastasis. These significantly influence the treatment choice and prognostic evaluation of patients.

The study tracked all records of PIML patients in the SEER database from 1979-2019, building a prognostic model over a forty-year cohort. This model is reliable in terms of sample size. And it can provide a basis for subsequent multicenter clinical studies or prospective studies. At the same time, imaging information was missing from the data we collected, and this absence is understandable. The first publication on ^18^F FDG PET in lymphoma was in 2007 (32). Also, medical imaging system is evolving very rapidly, it is difficult to document with quantifiable information, and imaging changes in patients at different pathological stages are dynamic. Therefore, this study focuses more on quantifiable factors in the data and uses different factors: gender, age, stage, and type of pathology to build models that predict the prognosis of different patients. Most of these factors can be obtained in the admission records, which is faster and more specific. Also imaging evidence can be used as valid evidence of prognosis, which, together with the results of this study, allows a better management and assessment of the prognosis and treatment of patients. In other words, with our prognostic model as a basis, we can combine imageomics and molecular diagnostics to build a multimodal prognostic model (35). It is a big challenge that requires more clinical studies and further molecular research.

In summary, this study obtained comprehensive clinical data from the SEER database and assessed the actual situation of individual patients from different perspectives, using uniform criteria and methods. This allowed us to comprehensively analyze the clinicopathological features of intracranial lymphomas. However, this study had several limitations. First, the treatment-specific information in the cohort was not sufficiently comprehensive, which prevented us from further targeting the benefits of different treatment. Due to the limitations of the database, we do not have access to a definitive treatment plan, which is the factor that has the greatest impact on patient prognosis. This includes the surgical procedure, the size and extent of tumor resection, the management of metastases including lymph nodes, and postoperative management. This information can be more specific and refined for assessing the prognosis of patients. At the same time, more large multicenter clinical trials are needed to explore the details of these treatment factors, which have positive implications for improving the prognosis of patients with intracranial lymphoma. Second, the SEER database contains qualitative or semi-quantitative data, and the statistical reliability was, to some extent, impaired. Finally, the nature of retrospective studies inevitably results in bias (36). Although our findings and new models require further in-depth studies, our results can provide new insights for treating patients with PIML and can assist oncology hematologists.

## Conclusion

We constructed a new nomogram to predict OS and CSS in patients with PIML. In addition, we found that age, surgery, chemotherapy, Ann Arbor staging, and histological type are independent risk factors for PIML. The identification of risk factors and construction of nomograms can provide new insights for patients with PIML and oncology hematologists, allowing doctors to make better choices during clinical decision-making.

## Data availability statement

The original contributions presented in the study are included in the article/supplementary material. Further inquiries can be directed to the corresponding author.

## Author contributions

ZY and FP had the idea and wrote the main manuscript texts. CF ,ZL and YZ performed the literature search and data analysis. YL, YD, and NL drafted and critically revised the work. All authors contributed to the article and approved the submitted version. 
